# The importance of life history and population regulation for the evolution of social learning

**DOI:** 10.1098/rstb.2019.0492

**Published:** 2020-06-01

**Authors:** Dominik Deffner, Richard McElreath

**Affiliations:** Max Planck Institute for Evolutionary Anthropology, Department of Human Behavior, Ecology and Culture, Leipzig, Germany

**Keywords:** social learning, life history, culture, evolution, demography, population regulation

## Abstract

Social learning and life history interact in human adaptation, but nearly all models of the evolution of social learning omit age structure and population regulation. Further progress is hindered by a poor appreciation of how life history affects selection on learning. We discuss why life history and age structure are important for social learning and present an exemplary model of the evolution of social learning in which demographic properties of the population arise endogenously from assumptions about *per capita* vital rates and different forms of population regulation. We find that, counterintuitively, a stronger reliance on social learning is favoured in organisms characterized by ‘fast’ life histories with high mortality and fertility rates compared to ‘slower’ life histories typical of primates. Long lifespans make early investment in learning more profitable and increase the probability that the environment switches within generations. Both effects favour more individual learning. Additionally, under fertility regulation (as opposed to mortality regulation), more juveniles are born shortly after switches in the environment when many adults are not adapted, creating selection for more individual learning. To explain the empirical association between social learning and long life spans and to appreciate the implications for human evolution, we need further modelling frameworks allowing strategic learning and cumulative culture.

This article is part of the theme issue ‘Life history and learning: how childhood, caregiving and old age shape cognition and culture in humans and other animals’.

## 1. Introduction

Humans are exceptionally reliant on culture. Theoretical models of the evolution of learning have brought considerable insight into the adaptive logic of culture and the conditions under which it can evolve [1–4]. But humans are exceptional in many ways, and this constellation of unusual traits must be explained in a unified framework. In particular, human adaptation is integrated with our special life history [5,6]. We owe our ecological success to a highly developed ability to learn from others, but we also exhibit a prolonged juvenile period, shorter inter-birth intervals, and an extended post-reproductive lifespan [7,8]. Human children are dependent on an extended network of carers and develop more slowly than other apes, but we nonetheless can have more of them, in shorter intervals. These traits are arguably unique to the genus *Homo* [9] and might have coevolved with our extensive reliance on cultural adaptations that allow adults to produce enough energy surplus to fuel this long and expensive development [10–12]. It is still unclear how and when a fully modern life history first appeared. Apes, in general, are characterized by long, slow life histories that are most likely an adaptation for dealing with uncertainty in juvenile recruitment [8]. It has been argued that a fully modern life history was certainly not present in *Australopithecus* [9] but evolved more recently as a mosaic of different features [13]. The high fertility of long-lived humans supported by our skill-intensive, socially learned foraging niche and substantial allomaternal care allows our species to have multiple dependent offspring at the same time resulting in a much greater potential for population growth and territorial expansion [14].

To understand the integrated role of culture in human adaptation, we need theoretical work that includes age structure and explicitly deals with different life-history dynamics. Age structure is not only an undeniable feature of human (and other animal) populations, it also has profound and often unforeseeable consequences for evolutionary dynamics. Lifetime reproductive success, for instance, is not an adequate measure of fitness anymore as soon as there is age structure, because timing of reproduction and other age-dependent strategies become important determinants of lineage growth rate [15,16]. The age structure and life-history system of a population is also expected to profoundly shape the informational environment learning strategies are responding to. Optimal learning strategies are expected to be age-dependent and we still need a formal life-history theory of learning that includes cultural transmission. How should individuals combine different sorts of information over the course of their lifetime and how does that affect the population distribution of cultural variants? Researchers have used dynamic optimization approaches to compute optimal learning schedules in response to a fixed set of environmental challenges [17,18], but including cultural transmission makes learning strategies inherently frequency-dependent, complicating the use of optimality approaches. Important first steps in this direction are the models by Aoki *et al.* [19,20] who solve for evolutionarily stable learning schedules and investigate which conditions result in cumulative cultural evolution. Similarly, Fogarty *et al*. [21] modelled how different sequences of dominant transmission modes throughout an individual’s lifetime affect evolutionary dynamics.

While including age-dependent learning is crucial in understanding how culture and life-history interact, there are also very basic open questions about the adaptive value of culture and of different social learning strategies in age-structured populations. Including age structure in models of social learning requires assumptions about vital rate parameters and the way population growth is regulated. What regulates population size has been described as ‘the fundamental question of population ecology’ [22]. Any population surviving and reproducing at a constant *per capita* rate will either go extinct or grow exponentially. Growth in natural populations is instead density dependent. Vital rates are said to be density dependent if they change depending on the density of conspecifics, owing to resource depletion or competition for territory [23,24]. Density-dependent population regulation acts as a negative feedback mechanism that keeps a population within the carrying capacity of its environment. Based on a large abundance time-series database covering 1198 species, Brook & Bradshaw [25] demonstrated that density dependence is indeed a pervasive feature of population dynamics in the wild that holds across widely different taxa. Density-independent factors, by contrast, such as natural disasters, weather or pollution, exert their influences on population size regardless of the population’s density and, thus, cannot keep a population at constant levels.

The way a population is regulated through density-dependent factors is known to shape the demographic structure of a population [24] and may change the incentives for copying or innovating even if the equilibrium population size and vital rates are constant. Starting with Henrich’s [26] model, that showed how reduced population sizes might have led to the loss of adaptive cultural knowledge in Tasmania, there is now a considerable literature on the importance of population size for cultural evolution [27–31]. It has also been suggested that connectivity or network structure plays a vital role in cultural dynamics [32–34].

Whatever the importance of population size and connectivity in cultural evolution, these features do not suffice to describe the constitution of a population, once demography and age structure are included. A given population size can result from numerous different constellations of vital rates and population regulation regimes which might exert different selection pressures on learning strategies. Vital rates and population regulation jointly determine the age structure of the population, influence when organisms die, when juveniles are born, and how much adaptive information the population possesses at these times. Importantly, even if researchers do not explicitly consider different vital rate constellations and population regulation regimes, they must make implicit assumptions about the way the population is maintained, the implications of which are poorly understood. With respect to the evolution of human life-history traits, for example, Baldini [35] demonstrated that the conclusions of an influential model do not hold if the implied mechanism of density dependence is changed.

This suggests an important project of reconsidering models of the evolution of social learning under different population-regulation and life-history scenarios. In this paper, we aim to clarify the impact of density-dependent population regulation and different life histories on the adaptive value of culture. We present a model of the evolution of social learning in which demographic properties of the population arise endogenously from assumptions about *per capita* vital rates and separate forms of population regulation, and compare the extent to which social information use is favoured under different scenarios. We find in even these simplest models—a necessary first step in building this literature—that paradoxical effects may arise, such as social learning evolving more readily when lifespan is short. We are able to explain these paradoxical effects in light of the costs and benefits of learning. We close by discussing limits of these models and future directions.

## 2. Methods

We constructed two models that differ only in the way population size is regulated, either through reduced chances of survival or through reduced chances of giving birth. First, we formulated analytical expressions of the basic population dynamics assuming only two age classes. These demographic models were then used to derive principled parameter combinations for individual-based simulations that allow comparisons of learning dynamics between different population-regulation and life-history regimes while holding other factors constant.

### (a) Population regulation

In the simplest models, populations under density-dependent regulation follow a logistic growth curve. Originally developed to model stock dynamics and recruitment in fisheries, Bill Ricker formulated a discrete-time equivalent to the continuous-time logistic model, commonly known as the Ricker map [36]:

2.1 $$N_{t+1}=N_{t}{\text{e}}^{r(1-(N_{t}/K))}.$$

*N*_*t*_ is the population size at time *t* and *K* gives the carrying capacity of the environment. Decomposing the population growth rate *r* into its components, birth rate and death rate, one can differentiate between regulation acting through increased mortality or regulation acting through decreased fertility. Although regulation in nature might often act through both mortality and fertility, we will focus on these pure types of density-dependent population regulation, which we will refer to as ‘mortality regulation’ and ‘fertility regulation’, respectively. Focusing on pure mechanisms helps to reveal causal impacts.

### (b) Model definitions

#### (i) Fertility regulation

In the first model, *per capita* fertility (probability of giving birth per individual per time unit) decreases as the population grows, whereas *per capita* survival (probability of survival per individual per time unit) is independent of population size. The population dynamics are captured by the following recursions that describe juvenile (class 0) and adult (class 1) individuals, respectively:

2.2 $$N_{0,t+1}=N_{1,t}b{\text{e}}^{-\delta N_{t}}$$

and

2.3 $$N_{1,t+1}=(N_{1,t}+N_{0,t})s.$$

The number of juveniles at the next time step, *N*_0,*t*+1_, results from the number of adults at present, *N*_1,*t*_, times the *per capita* fertility rate *b* which declines exponentially with larger population sizes leading to smaller chances of having offspring. The strength of this exponential decay is determined by the fertility regulation parameter *δ*. The number of adults at *t* + 1, *N*_1,*t*+1_, equals the number of adults that survive from the present time step plus the number of juveniles that are recruited to the breeding population (both with probability *s*).

#### (ii) Mortality regulation

In the second model, *per capita* survival rates decrease with increasing population size, while fertility remains constant:

2.4 $$N_{0,t+1}=N_{1,t}b$$

and

2.5 $$N_{1,t+1}=(N_{1,t}+N_{0,t})s{\text{e}}^{-\gamma N_{t}}.$$

Again, the number of juveniles at the following time step equals the number of adults at present times the (now density independent) *per capita* fertility rate *b*. Adults in the following time step are all adults that survive from the present time step plus the juveniles that survive into adulthood. In this model, survival rates now decrease exponentially as population size increases. The strength of this decrease is determined by the mortality regulation parameter *γ*.

Both modes of regulation lead to a similar logistic population growth curve that flattens out at equilibrium population size $\hat{N}$ (see the electronic supplementary material, figure S1). It can be shown (see the electronic supplementary material for details) that the equilibrium population size under fertility regulation is given by

2.6 $${\hat{N}}_{\mathrm{Fertility}}=\frac{\log((1-s)/bs)}{-\delta },$$

and the equilibrium population size under mortality regulation is given by

2.7 $${\hat{N}}_{\mathrm{Mortality}}=\frac{\log(1/s(1+b))}{-\gamma }.$$

Figure S2 in the electronic supplementary material illustrates how vital rate parameters (*s* and *b*) and the respective regulation parameter (*δ* or *γ*) jointly determine the equilibrium population size in this model. We make the simplifying assumption that baseline vital rates are constant over the lifespan, with the exception that juveniles do not yet reproduce. Implementing more primate-typical mortality and fertility patterns could further clarify the implications of density dependence in primate evolution [37].

### (c) Derivation of principled parameter values

Based on these analytical expressions, we found principled combinations of vital rate and regulation parameters that allow a direct evaluation of their effect on the evolution of learning while keeping other factors constant. We wanted to cover a broad range of different life histories, represented by distant points on the isoclines in electronic supplementary material, figure S2, to explore how population regulation and vital rate parameters jointly affect selection on social learning.

We chose different values for the equilibrium population size $\hat{N}$ (200, 350, 500), the expected lifespan $\hat{L}$ (3, 5, 7.5) and fertility regulation parameter *δ* ($\frac{1}{550},\frac{1}{1000},\frac{1}{1500}$) and derived all other parameter values for both modes of regulation (see the electronic supplementary material for details). We will refer to constellations with relatively high mortality and fertility rates as ‘fast’ life histories and to constellations with relatively low mortality and fertility rates as ‘slow’ life histories [38–40].

### (d) Social learning simulations

Building on the demographic models, we constructed individual-based simulations to explore the consequences of different life-history constellations on the evolution of social learning in stochastically changing environments. The simulation tracks the behaviour of each individual in a single age-structured population of varying, but finite, size through the sequence of birth and mutation, learning, mortality and ageing, and environmental stochasticity. We focus on one domain of behaviour for which there is a single adaptive variant for any state of the environment. Possessing this adaptive variant increases individuals’ chance of survival by a factor *σ* > 1 and their chance of reproduction by a factor *β* > 1. As explained in the previous section, equilibrium population sizes, $\hat{N}$, were calculated based on baseline fertility and survival rates. Thus, actual population sizes in the simulations vary depending on the proportion of adapted individuals and exceed the $\hat{N}$ from the analytical solutions. We assume an infinite state environment that never reverts to an earlier state. This implies that individuals can acquire the adaptive variant only through learning.

#### (i) Birth and mutation

We assume asexual, haploid reproduction. At the beginning of each time step, all adult individuals give birth to a single offspring with probability *b* (non-adapted) and probability *βb* (adapted), respectively. Under fertility regulation, these rates are multiplied by ${\text{e}}^{-\delta N_{t}}$ to make them density dependent. Juveniles inherit a learning parameter *ξ* that deviates slightly from their parent’s value in a random direction. Specifically, during each mutation event a value drawn from $N(0,\mu_{\xi })$ is added to the value of their parent while ensuring that the resulting *ξ* value remains within the interval [0,1].

#### (ii) Learning

All juveniles have the opportunity to acquire the adaptive variant through learning, either individually or socially. Specifically, a juvenile learns individually with probability *ξ* and socially with probability 1 − *ξ*. As learning strategies in nature are most likely influenced by myriads of different genes, *ξ* can be thought of as their cumulative effect that expresses an individual’s tendency towards individual learning. If an individual learns socially, it copies the variant of a randomly chosen adult. If it learns individually, it has a chance *w* to invent the adaptive solution. Letting only juveniles learn is clearly unrealistic for any real organism, but making simplifying assumptions is critical for understanding complex systems [41]. Allowing only juveniles to learn represents the extreme case of the exploration–exploitation trade-off organisms face between investing in learning as opposed to allocating their time and energy to reproduction.

#### (iii) Survival and ageing

After learning occurred, all individuals must survive. For both juveniles and adults, there is a chance *s* (non-adapted) and *σs* (adapted), respectively, that they survive until the next time step. Under mortality regulation, these rates are multiplied by ${\text{e}}^{-\gamma N_{t}}$. Juveniles that learned individually pay a once-only survival cost *c* that reduces their chance to survive into adulthood. This reflects the commonly held assumption that individual learning is more costly than copying, as individuals may spend considerable amounts of time and resources independently exploring the environment [1,2].

#### (iv) Environmental stochasticity

After each time step, there is a probability *u* that the environment changes. When environmental change occurs, all variants in the population become non-adaptive.

See the electronic supplementary material, table S1 for a summary of all parameters used in the simulations. We compared equilibrium population sizes of 200, 350 or 500 individuals, expected lifespans of 3, 5 and 7.5 years (corresponding to $s=0.6\bar{6}$, 0.8, $0.8\bar{6}$) and weak ($\delta =\frac{1}{1500}$), moderate ($\delta =\frac{1}{1000}$) and strong fertility regulation ($\delta =\frac{1}{550}$) and derived the respective vital rate and mortality regulation parameters from the expressions introduced in the previous section. These values were chosen to represent the widest possible range of population sizes and life histories that were compatible with the architecture of our analytical model. We also systematically varied the rate of environmental change *u* (every 10th, 100th or 1000th time step), the cost of individual learning *c* (1%, 5%, 10% reduced chance of surviving into adulthood) and the success rate of individual learning *w* (1%, 10%, 50%, 90% and 99%). All simulations were programmed in R [42]. Simulation and plotting code can be found on GitHub: https://github.com/DominikDeffner/LifeHistorySocialLearning.

## 3. Results

All results reported in the following come from the last 5000 time steps of 10 independent 7000 time-step simulations per parameter combination. This duration was sufficient to reach steady state in all cases. In the main text, we report results for moderate strength of population regulation ($\delta =\frac{1}{1000}$). Results for stronger or weaker regulation were very similar and can be seen in the electronic supplementary material, figures S6 and S7.

### (a) Demographics and adaptation dynamics

Electronic supplementary material, figure S3 illustrates the basic demographics and adaptation dynamics for one exemplary parameter combination. Right after a switch in the environment (indicated by dashed lines), all individuals become non-adapted and the population size declines. As only juveniles learn, adaptation levels start to increase earlier in younger age classes compared to older age classes. Five years after the environment has changed, for instance, only individuals aged 5 or younger might possess the adaptive variant, whereas older individuals have learned before the environment has changed. Long after an environmental change, population size fluctuates around the carrying capacity and the proportion of adapted individuals tends to be higher in older age classes; selection functions as a population filter and those possessing the adaptive variant are more likely to survive to old ages.

### (b) Lifespan

**Figure 1.**
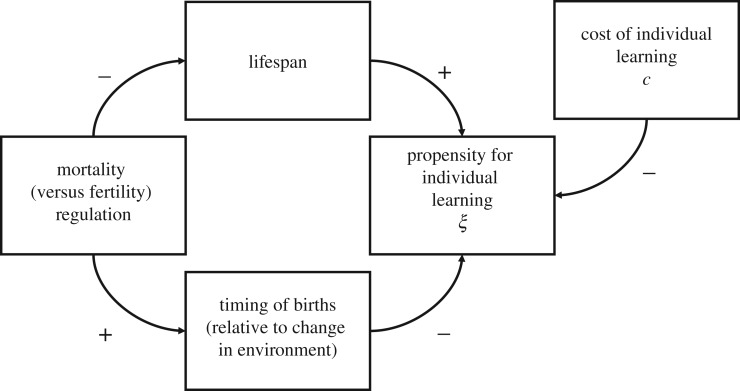
Life history and demographic forces influencing selection on learning.

**Figure 2.**
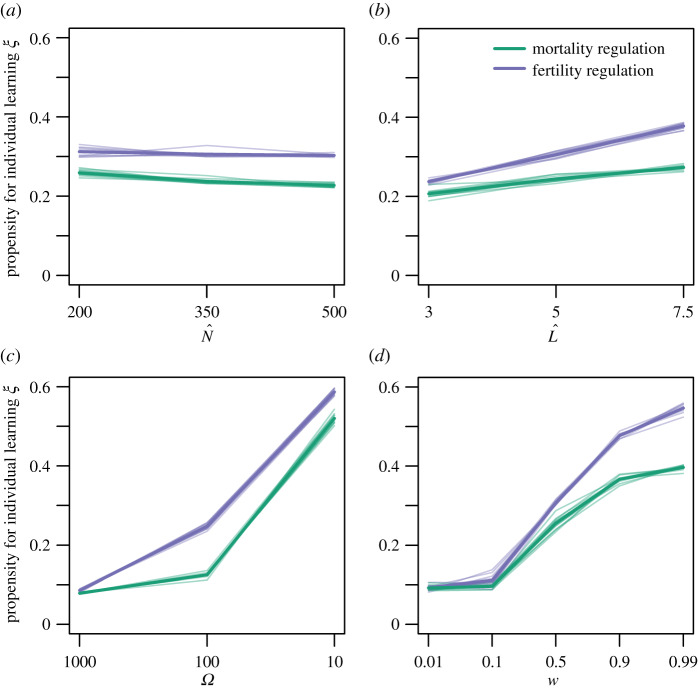
Average propensity for individual learning *ξ* as a function of (*a*) equilibrium population size $\hat{N}$ (values are based on baseline vital rates and thus correspond to situations when all individuals are not adapted); (*b*) expected lifespan $\hat{L}$ (values of 3, 5 and 7.5 years correspond to $s=0.6\bar{6}$, *s* = 0.8 and $s=0.8\bar{6}$, respectively); (*c*) expected time between environmental changes *Ω* ($=\frac{1}{u}$); and (*d*) success rate of individual learning *w*. Transparent lines show results from 10 independent simulations, solid lines represent averages across different simulations. Results are averaged over all values of other parameters (*c* = 0.95). (Online version in colour.)

Figure 1 shows a conceptual diagram of the main demographic forces that influence selection on learning in this model. Slower life histories, characterized by long lifespans and low fertility rates, resulted in more individual learning compared to faster life histories (figure 2*b*). It is counterintuitive that it is fast life histories that favour more social information use instead of the slow life histories typical of primates. However, by determining how long individuals live, $\hat{L}$ influences the relative length of generation time and expected time between environmental changes [43]. If lifespans are long, conditions are more likely to change within generations and many adults will not have learned since the last switch in the environment. In this case, it pays for a juvenile to learn individually. Also, longer lifespans make early investments in learning more profitable, as organisms have more opportunities to reproduce later on. As individual learning is assumed to be more costly than copying, it is favoured when organisms live long enough to make up for their early investment in learning. This is confirmed by simulations with different costs of individual learning: lowering the recruitment cost to just 1% (as opposed to 5%) largely removed the effect of lifespan on social information use, whereas increasing it to 10% amplified the difference (electronic supplementary material, figures S4 and S5).

### (c) Population regulation

Population regulation through increased mortality consistently favoured stronger reliance on social learning compared to regulation through reduced fertility. This effect of population regulation was particularly strong in simulations with long lifespans, intermediate to fast changing environments and relatively high success rates of individual learning (figure 2).

There are two mechanistic pathways that explain the effect of population regulation on learning (figure 1). If baseline vital rates are constant, mortality regulation will necessarily result in shorter lifespans compared to fertility regulation. We have seen before how shorter lifespans result in more social learning in the present model. In line with the reasoning that individual learning involves a trade-off between lower juvenile survival and the potential for higher lifetime reproduction, the cost of individual learning modulates the effect of population regulation on learning. If individual exploration is essentially costless (*c* = 0.01), the difference between populations that are regulated through mortality and fertility, respectively, is much smaller compared to simulations with higher costs of individual learning (electronic supplementary material, figures S4 and S5).

**Figure 3.**
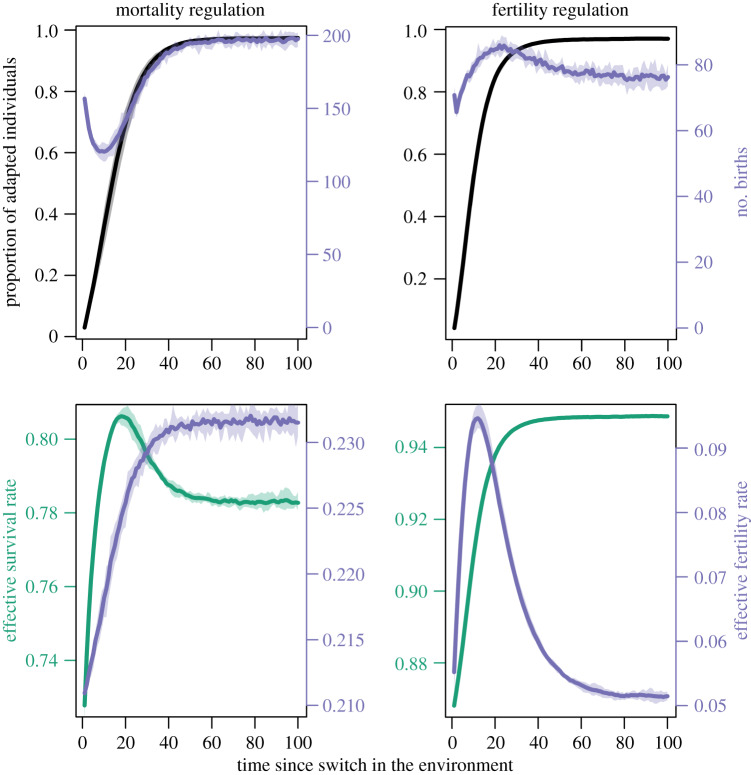
Effect of population regulation on timing of reproduction. The top row shows proportion of adapted individuals in black and number of juveniles in purple; the bottom row displays effective fertility rates in purple and effective survival rates in green; mortality regulation is shown on the left, fertility regulation on the right. Shaded areas represent variation across means of 10 independent simulations. Plot shown for $\hat{N}=500$, $\hat{L}=7.5$, $\delta =\frac{1}{1000},\sigma =1.1$, *β* = 1.1, *u* = 0.01,*w* = 0.9, *c* = 0.05 and $\mu_{\xi }=0.01$. (Online version in colour.)

The second pathway is through the influence of regulation on the timing of reproduction (figure 3). Under mortality regulation, birth numbers (purple) drop after a change in the environment and reach their highest level when almost all adults are adapted. For those juveniles, it is likely to be adaptive to learning socially. Under fertility regulation, by contrast, many juveniles are born relatively shortly after the environment has changed, when a substantial proportion of adults do not possess the adaptive variant and it might be more beneficial to learn individually. The bottom row of figure 3 displays the trajectories for effective vital rates, the actual per-individual probabilities of surviving (green) and reproducing (purple) at any point in time (see the electronic supplementary material for details). Under mortality regulation, effective fertility rises as the proportion of adapted adults increases, resulting in most juveniles being born long after environmental changes, when most adults possess adaptive behaviour. The drop in actual birth numbers after an environmental change is owing to the decline in population size. Under fertility regulation, it is the survival probability that increases with the amount of adaptive knowledge. Fertility first also increases before—owing to density-dependent factors—sharply declining as population size rises. This demographic constellation of relatively many births when the environment has just changed favours more individual learning.

## 4. Discussion

Life history and age structure matter for the evolution of social learning and most previous models decided to leave out the complexities of real-world demography. Of course, making simplifying assumptions is critical to understand complex systems [41,44], but if we want to understand how culture evolves in real animals, it is not enough to study the dynamics of learning and cultural information in isolation. Instead, we need modelling frameworks incorporating real life history and demography that will help shed light on the question of how culture and life history interacted in shaping who we are. We are just starting to understand how combined life-history/social learning systems might behave and how they could be modelled. As a first step in this direction, we used a combination of demographic models and social learning simulations to investigate how different life cycles and forms of population regulation affect selection on learning. We found that, counterintuitively, a stronger reliance on social learning is favoured in organisms characterized by ‘fast’ life histories with high mortality and fertility rates compared to ‘slower’ life histories typical of primates. Results also unveiled greater social information use in populations that are regulated through mortality, compared to populations that are regulated through fertility. Vital rates and population regulation jointly influence when most juveniles are born, how long individuals live and when they are more likely to die. These demographic variables then influence the incentives to copy or to innovate.

As in Rogers’ influential model [2], social learning in the present model is parasitic, i.e. social learners scrounge adaptive information from individual learners who paid a cost to produce it [45,46]. We chose this relatively simple form of social learning to establish how population regulation and life-history dynamics can affect selection on learning in a well-understood modelling framework. In order for culture to increase population fitness, however, it must make individual learning either more accurate or less costly [47] and both empirical and theoretical results suggest that organisms usually do not copy indiscriminately but use a diverse set of learning strategies in different ecological and social contexts [4]. In this model, organisms could acquire adaptive behaviour through individual learning or one-shot interactions with a demonstrator at the beginning of their lives. Many essential skills in real animals, however, take generations to evolve and years to develop, which is particularly true for complex and causally opaque human culture [5,48]. If adaptive behaviour takes time and practice to develop, longer lifespans should allow individuals to reach higher skill levels and to fully capitalize on cultural information. Moreover, with cumulative culture, adaptive behaviour typically cannot be invented by single individuals on their own, so social learning should be necessary [6,49].

Although our finding of more social learning in short-lived organisms might appear unintuitive to some readers given the opposite empirical association observed in humans and other animals, our results should not be regarded as ‘negative’. The goal of theoretical modelling is not necessarily to reproduce empirical findings, but to sharpen our questions and clarify the implications of certain assumptions about natural processes [44]. Our model demonstrates that simple Rogers-style social learning can be very successful in short-lived organisms and does not explain the coevolution of long lifespans and social information use providing an important baseline for future studies. Such models should investigate the interplay between learning and life-history dynamics with more elaborate learning strategies that let organisms flexibly respond to different cues throughout ontogeny [47] and allow for cumulative cultural evolution [49], instead of the binary ‘adapted/not-adapted’ proposition used here. Important assumptions of the present model are also that learning only occurs in juveniles and the environment never reverts to an earlier state. If adults could repeatedly update their behaviour based on environmental cues and/or the environment could switch back to conditions only experienced by older individuals, older age classes can serve as a reservoir of adaptive information likely to increase the value of culture in long-lived organisms. The present model can be regarded as a cultural ‘null model’ nominating further analyses that will help us determine which additional aspects of cultural adaptation are necessary or sufficient to create selection for slower life histories. In a recent model without cultural transmission, Ratikainen & Kokko [50] found that plasticity does not only evolve in response to a given life history but that plasticity itself can shift the balance in the trade-off between survival and reproductive effort to favour greater longevity (see [51–53] for other work on life history and value of asocial learning). Similar models incorporating social learning and cumulative culture will be crucial in uncovering the multiple trade-offs involved in cultural adaptation.

Our model also suggests that the human mode of cultural adaptation characterized by slow development and long lifespans might not be the only, and probably not even the most common, form of adaptation through social learning and we should expect to see at least simple forms of social learning in many short-lived organisms. This result is in line with the accumulating empirical evidence for the prevalence of social information use in very short-lived organisms such as fruit flies [54,55] and bumblebees [56,57]. Danchin *et al*. [54], for example, used a transmission chain experiment to show that neutral traits can indeed become cultural markers of mate quality in *Drosophila*. Similarly, cephalopod molluscs evolved complex brains and high behavioural flexibility together with fast life histories, challenging the idea that intelligence necessarily coevolves with slow life history [58].

Understanding the coevolutionary relationships between social learning and life history will benefit both sides, cultural evolution and life-history theory. Including age structure and life history into models of social learning profoundly changes the informational landscapes learners are navigating, and social learning, on the other hand, can alter life-history trade-offs in ways that are unintelligible without taking culture into account.

## Supplementary Material

Mathematical derivations and supplemental figures

## Data accessibility

This article does not contain any new data. Simulation code can be found on GitHub: https://github.com/DominikDeffner/LifeHistorySocialLearning
