# Supplementary material for: The importance of life history and population regulation for the evolution of social learning
Source: Philos Trans R Soc Lond B Biol Sci. 2020 Jun 1;375(1803):20190492. doi: 10.1098/rstb.2019.0492 (PMC7293155; doi:10.1098/rstb.2019.0492)
Supplement: Mathematical derivations and supplemental figures [file rstb20190492supp1.pdf]

SUPPLEMENTARY MATERIAL FOR "THE IMPORTANCE OF LIFE HISTORY AND POPULATION REGULATION FOR THE EVOLUTION OF SOCIAL LEARNING"

DOMINIK DEFFNER[1] & RICHARD MCELREATH[1]

[1] *Max Planck Institute for Evolutionary Anthropology, Department of Human Behavior, Ecology and Culture.*

Summary
Appendix (A) describes the derivation of equilibrium population sizes from the model recursions for both modes of population regulation; (B) shows how principled simulation parameter combinations were derived from analytical expressions; (C) explains how effective vital rate parameters in Fig. 3 were calculated; (D) summarizes all parameters and values used in the simulation models and (E) contains further results and illustrations.

A. Derivation of equilibrium population sizes

Fertility Regulation
Recall that the number of adults and juveniles under fertility regulation are given by the following recursions, respectively:

$$N_{0,t+1} = N_{1,t} b e^{-\delta N_t} \tag{S1}$$

$$N_{1,t+1} = (N_{1,t} + N_{0,t}) s \tag{S2}$$

Assuming we are at equilibrium and the population has reached its stable age distribution, we can insert the numbers of juveniles at equilibrium given by equation S1 into equation S2 which yields:

$$\hat{N}_1 = \hat{N}_1 s + \hat{N}_1 b e^{-\delta \hat{N}} s \tag{S3}$$

Solving this equation for $\hat{N}$ yields an expression for population equilibrium under fertility regulation.

Mortality Regulation
The number of adults and juveniles under mortality regulation are given by the following recursions:

$$N_{0,t+1} = N_{1,t}b \tag{S4}$$

$$N_{1,t+1} = (N_{1,t} + N_{0,t})se^{-\gamma N_t} \tag{S5}$$

Following the same strategy as above, we first insert the numbers of juveniles at equilibrium given by equation S4 into equation S5 which yields:

$$\hat{N}_1 = \hat{N}_1 se^{-\gamma\hat{N}} + \hat{N}_1 bse^{-\gamma\hat{N}} \tag{S6}$$

Solving for $\hat{N}$, we have the expression for equilibrium population size under mortality regulation.

B. Derivation of parameter combinations

We started by finding vital rate parameter values that result in the chosen $\hat{N}$ and $\hat{L}$ under the specified strength of fertility regulation and, then, solved for the unique value of mortality regulation parameter γ that gives the same $\hat{N}$ for the same s and b. If baseline vital rates are constant across different forms of population regulation, expected life spans will necessarily be shorter under mortality regulation, as per-capita survival is reduced for any positive population size. Under fertility regulation, s is simply given by:

$$s = \frac{\hat{L} - 1}{\hat{L}} \tag{S7}$$

while the corresponding fertility rate b can then be calculated as follows:

$$b = \frac{1 - s}{se^{-\delta\hat{N}}} \tag{S8}$$

This gives the unique combination of s and b that yield the specified equilibrium population size given the strength of fertility regulation. A relatively short expected life span of $\hat{L} = 3$ years, for instance, is realized through a per-time-step survival probability of $s = 0.6\bar{6}$. Under relatively weak fertility regulation ($\delta = \frac{1}{1500}$; Fig. S2A), this would require a fertility rate of 0.61 to produce an equilibrium population size of 300. Under stronger regulation ($\delta = \frac{1}{550}$; Fig. S2B), b would have to be 0.86 to produce the same population size. A relatively long expected life span of 7.5 years, in contrast, is realized through a per-time-step survival probability of $s = 0.8\bar{6}$. As fewer individuals die, comparatively lower fertility rates will be required to result in the same $\hat{N}$. With weak fertility regulation, for example, $b = 0.188$ will suffice to produce the same $\hat{N}$ of 300.

To find an equivalent value for the mortality regulation parameter γ that results in the same equilibrium population size under the same constellation of s and b, we

equated expressions 2.6 and 2.7 and solved for γ, which gives:

$$\gamma = \delta \frac{\log(\frac{1}{s(1+b)})}{\log(\frac{1-s}{bs})} \tag{S9}$$

The fraction, which is multiplied by δ, evaluates to a positive number and simply determines the slope of a line that, for any δ, gives the unique value of γ that results in the same $\hat{N}$.

C. Effective vital rates

The actual number of births is the result of many interacting factors, such as population size, vital rates and the proportion of adapted adults. Teasing apart these factors, we calculated effective vital rates for different times after an environmental change. Effective vital rates represent the actual per-individual probability of surviving or reproducing at any point in time and depend on baseline vital rates, population size, proportion of individuals (and adaptive behaviors) in different age classes and mean propensities for individual learning.

The effective fertility rate at timestep i after an environmental change is calculated as follows:

$$b_{\text{eff},i} = J_i 0 + (1 - J_i)(q_{A,i}\beta + (1 - q_{A,i}))be^{-\delta N_i} \tag{S10}$$

J_i gives the proportion of the population that are juveniles, $q_{A,i}$ represents the proportion of adults that possess adaptive behavior, b is the baseline fertility, β is the fertility advantage for adapted individuals, δ is the parameter controlling fertility regulation and N_i is the average population size. The effective survival rate, on the other hand, is given by:

$$\begin{aligned} s_{\text{eff},i} =& J_i((1 - \xi_{J,i})(q_{J,i}\sigma + (1 - q_{J,i}))se^{-\gamma N_i} \\ &+ \xi_{J,i}(q_{J,i}\sigma + (1 - q_{J,i}))cse^{-\gamma N_i}) \\ &+ (1 - J_i)(q_{A,i}\sigma + (1 - q_{A,i}))se^{-\gamma N_i} \end{aligned} \tag{S11}$$

$\xi_{J,i}$ represents the average propensity for individual learning among juveniles, $q_{J,i}$ gives the proportion of juveniles that are adapted, c is the recruitment cost of individual learning, s indicates baseline survival probability, σ is the survival advantage for adapted individuals and γ is the mortality regulation parameter.

D. Summary of parameters and values used in simulation models

Table S1. Summary of simulation parameters

**Parameter**	**Values**	**Description**
$\hat{N}$	$\{200, 350, 500\}$	Equilibrium population size if non-adapted
$\hat{L}$	$\{3, 5, 7.5\}$	Expected life span under fertility regulation
s	$f(\hat{L}) \in \{0.6\bar{6}, 0.8, 0.8\bar{6}\}$	Baseline per-capita survival rate
b	$f(s,\, \hat{N},\, \delta) \in [0.176,\, 0.824]$	Baseline per-capita fertility rate
σ	1.1	Survival advantage for adapted individuals
β	1.1	Fertility advantage for adapted individuals
ξ	[0,1]	Probability of individual learning
μ_ξ	0.01	Mutation rate for ξ
δ	$\{\frac{1}{550}, \frac{1}{1000}, \frac{1}{1500}\}$	Fertility regulation parameter
γ	$f(s,\, b,\, \delta) \in [9.4\text{-}05,\, 3.9\text{e-}04]$	Mortality regulation parameter
c	$\{0.01, 0.05, 0.1\}$	Recruitment cost of individual learning
w	$\{0.01, 0.1, 0.5, 0.9, 0.99\}$	Success rate of individual learning
u	$\{0.001, 0.01, 0.1\}$	Probability of environmental change

E. Extended Results

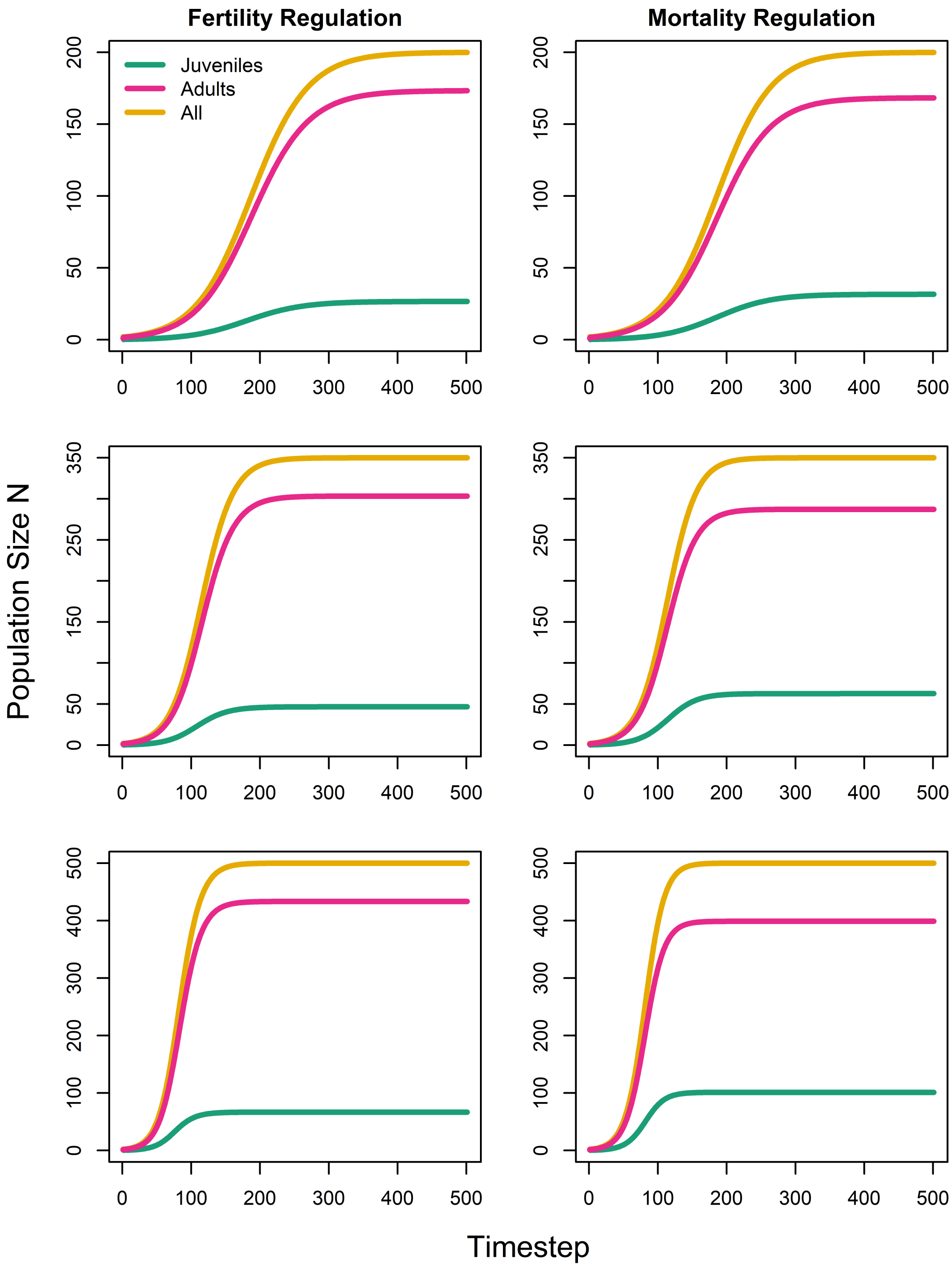

FIGURE S1. Population growth curves for adults (pink), juveniles (green) and both combined (yellow) according to analytical models; fertility regulation on the left and mortality regulation on the right; plots show trajectories for equilibrium population sizes of 200, 350 and 500, respectively (from top to bottom). $\delta = 1/1000$, $\hat{L} = 7.5$

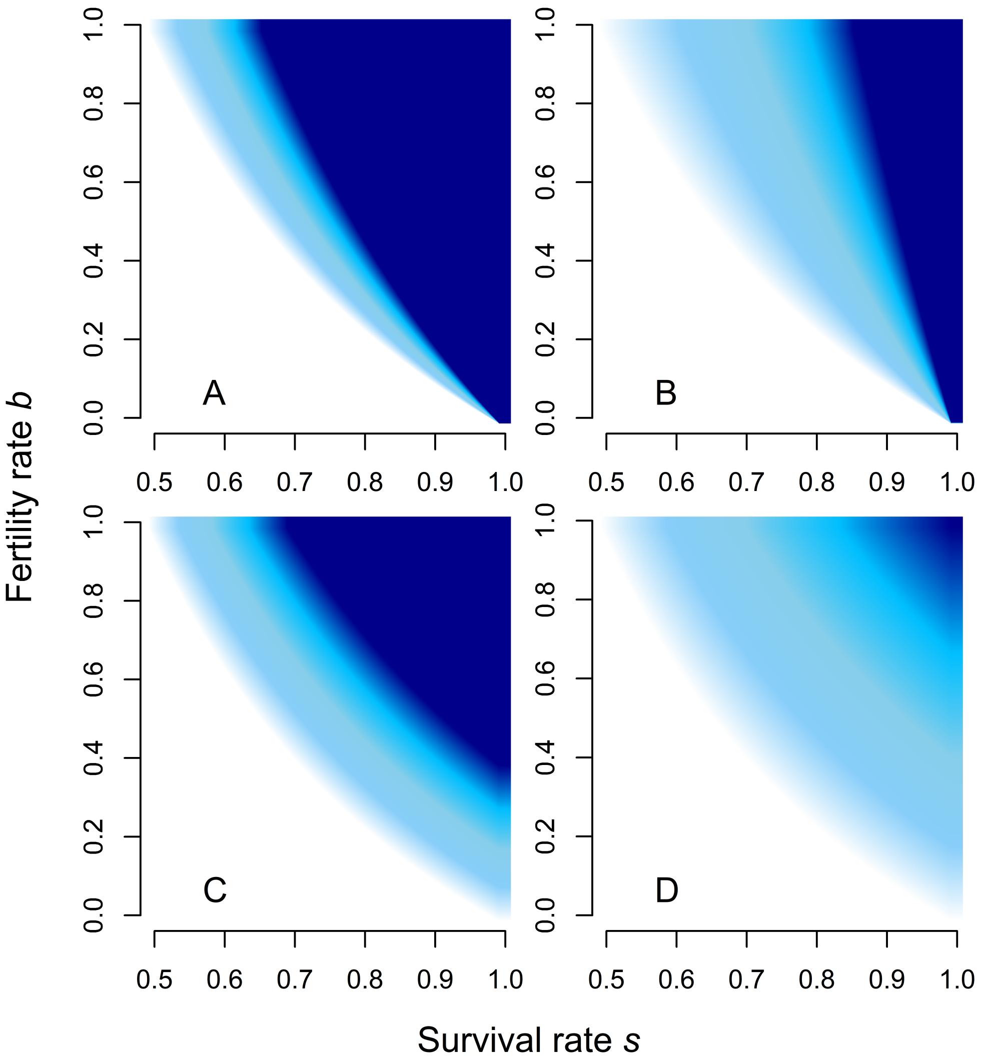

FIGURE S2. Isoclines for different equilibrium population sizes; parameter constellations resulting in non-viable populations are drawn in white and those resulting in population sizes of $\hat{N} > 1000$ are drawn in dark blue. Each curve gives combination of survival and fertility rates that produce the same equilibrium population size; the curve for $\hat{N} = 0$ corresponds to the vital rates that produce a stationary population if there is no population regulation. Survival rates s are only plotted for the interval $[0.5, 1]$, as lower survival rates will never result in a viable population. (A) Weak fertility regulation ($\delta = \frac{1}{1500}$); (B) Strong fertility regulation ($\delta = \frac{1}{550}$); (C) Weak mortality regulation ($\gamma = \frac{1}{3000}$); (D) Strong mortality regulation ($\gamma = \frac{1}{1000}$).

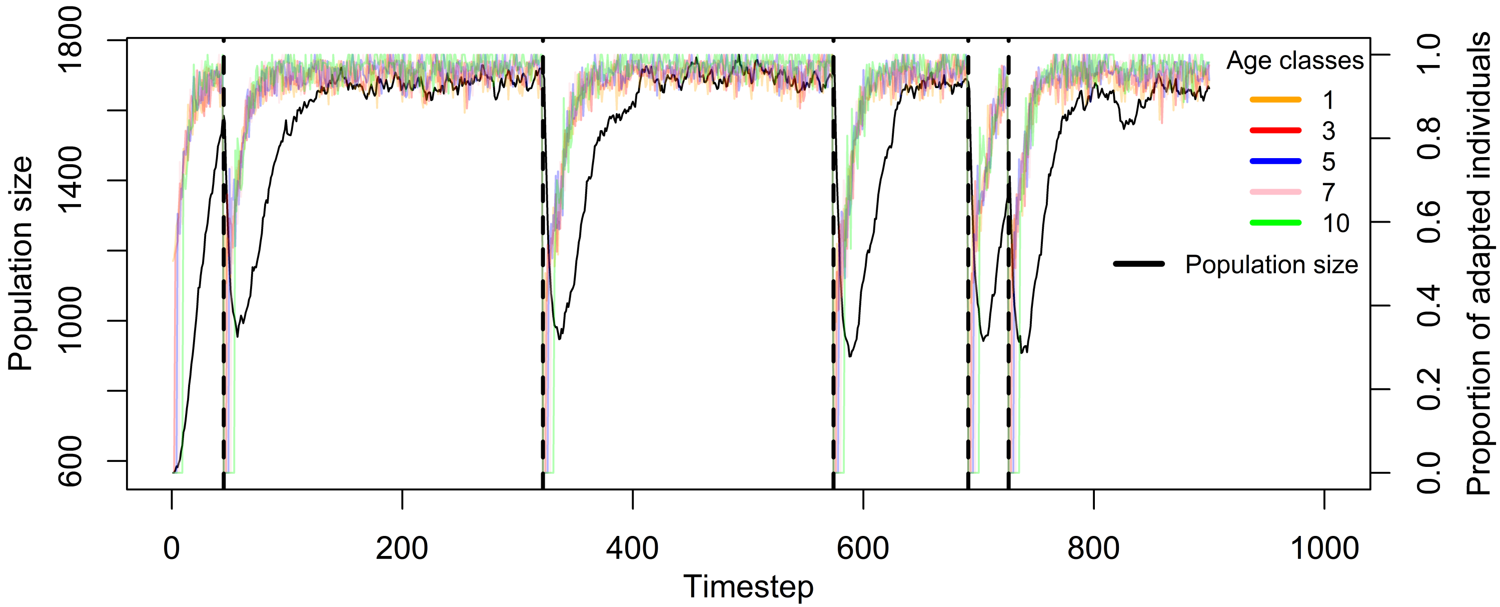

FIGURE S3. Illustration of basic demographics and adaptation dynamics. Vertical dashed lines represent times when the environment switched; the black curve represents population size and colored curves represent the proportion of adapted individuals in selected age classes at each point in time. Plot shown for $\hat{N} = 500$, $\hat{L} = 7.5$, $\delta = \frac{1}{1000}$, $\sigma = 1.1$, $\beta = 1.1$, $u = 0.01$, $w = 0.9$, $c = 0.05$ and $\mu_\xi = 0.01$.

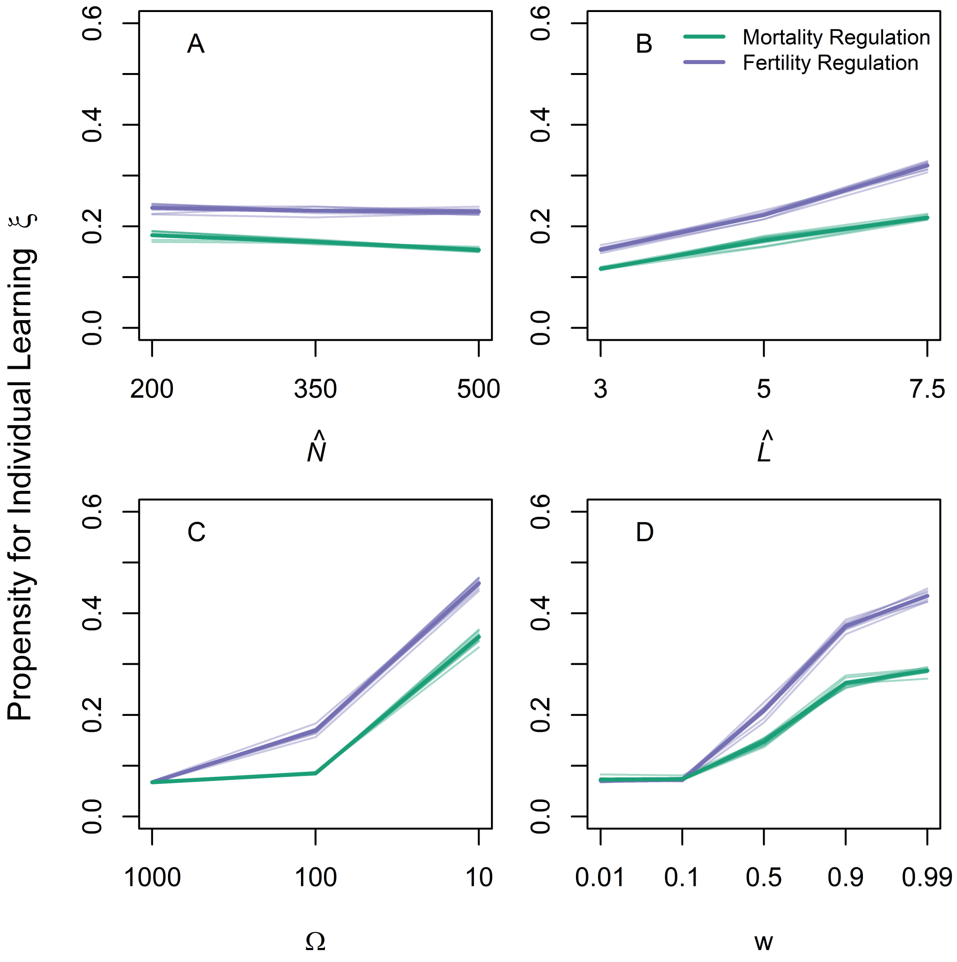

FIGURE S4. Results for simulations with high cost of individual learning ($c = 0.1$). Average propensity for individual learning ξ as a function of (A) equilibrium population size $\hat{N}$ (values are based on baseline vital rates and thus correspond to situations when all individuals are not adapted); (B) expected life span $\hat{L}$ (values of 3, 5 and 7.5 years correspond to $s = 0.6\bar{6}$, $s = 0.8$ and $s = 0.8\bar{6}$, respectively) ; (C) expected time between environmental changes Ω ($= \frac{1}{u}$) and (D) success rate of individual learning. Transparent lines show results from 10 independent simulations, solid lines represent averages across different simulations. Results are averaged over all values of other parameters.

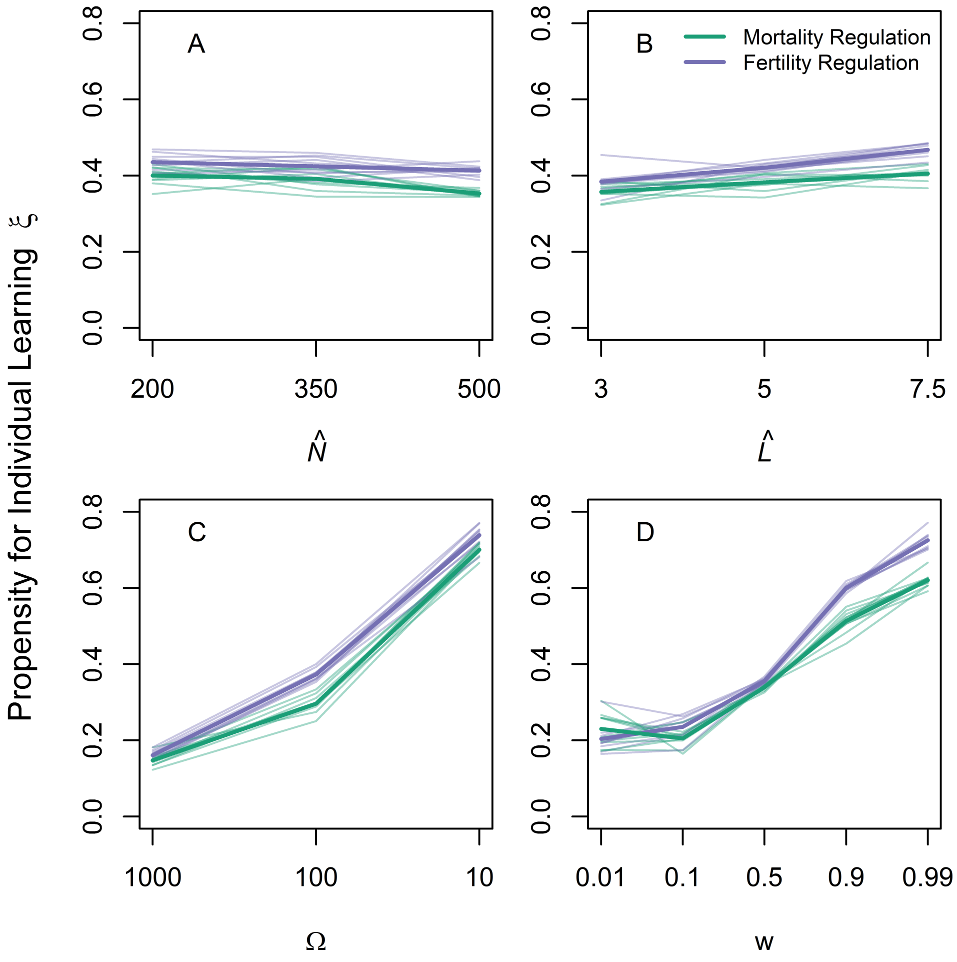

Figure S5. Results for simulations with low cost of individual learning ($c = 0.01$). Average propensity for individual learning ξ as a function of (A) equilibrium population size $\hat{N}$ (values are based on baseline vital rates and thus correspond to situations when all individuals are not adapted); (B) expected life span $\hat{L}$ (values of 3, 5 and 7.5 years correspond to $s = 0.6\bar{6}$, $s = 0.8$ and $s = 0.8\bar{6}$, respectively) ; (C) expected time between environmental changes Ω $(= \frac{1}{u})$ and (D) success rate of individual learning. Transparent lines show results from 10 independent simulations, solid lines represent averages across different simulations. Results are averaged over all values of other parameters.

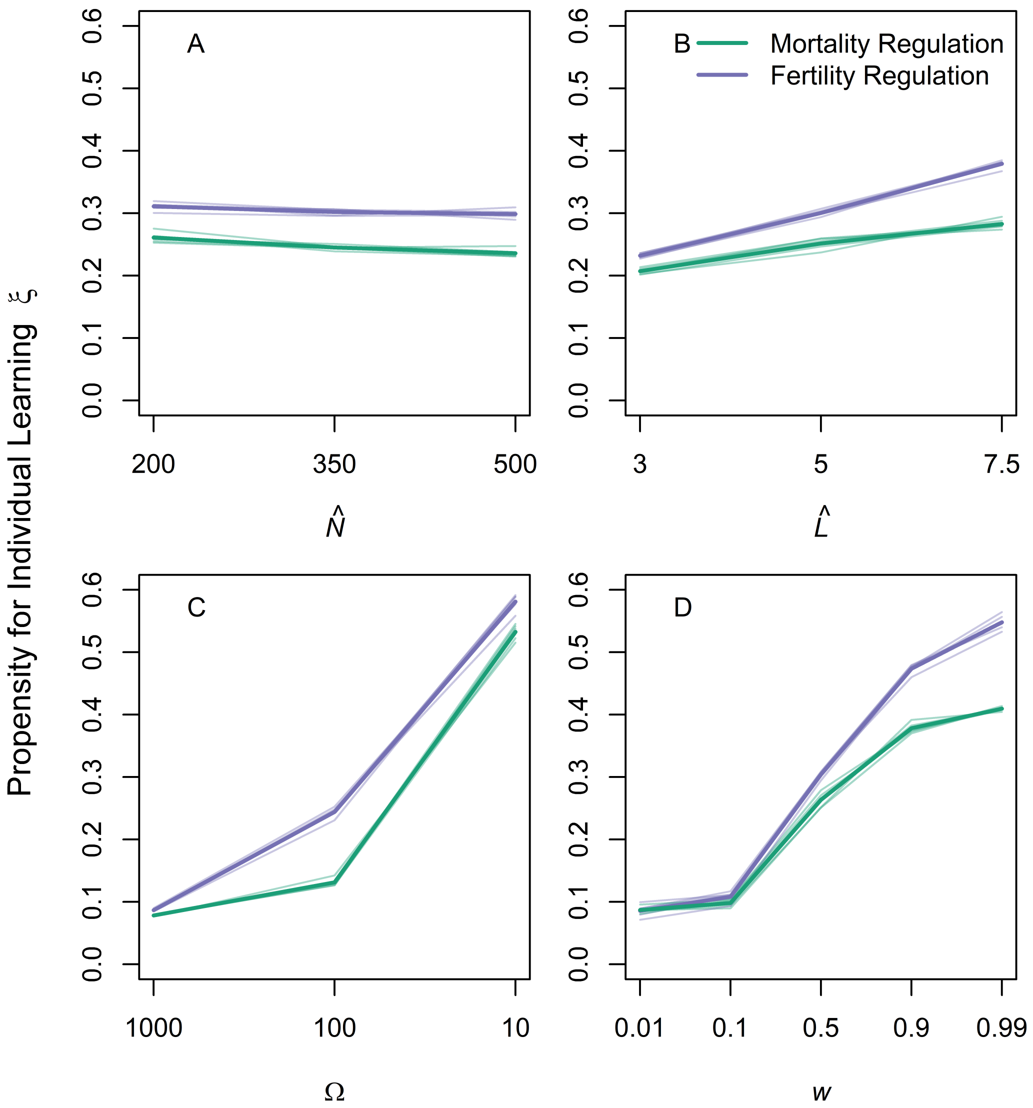

Figure S6. Results for simulations with weak regulation ($\delta = \frac{1}{1500}$). Average propensity for individual learning ξ as a function of (A) equilibrium population size $\hat{N}$ (values are based on baseline vital rates and thus correspond to situations when all individuals are not adapted); (B) expected life span $\hat{L}$ (values of 3, 5 and 7.5 years correspond to $s = 0.6\bar{6}$, $s = 0.8$ and $s = 0.8\bar{6}$, respectively) ; (C) expected time between environmental changes Ω ($= \frac{1}{u}$) and (D) success rate of individual learning. Transparent lines show results from 5 independent simulations, solid lines represent averages across different simulations. Results are averaged over all values of other parameters ($c = 0.95$).

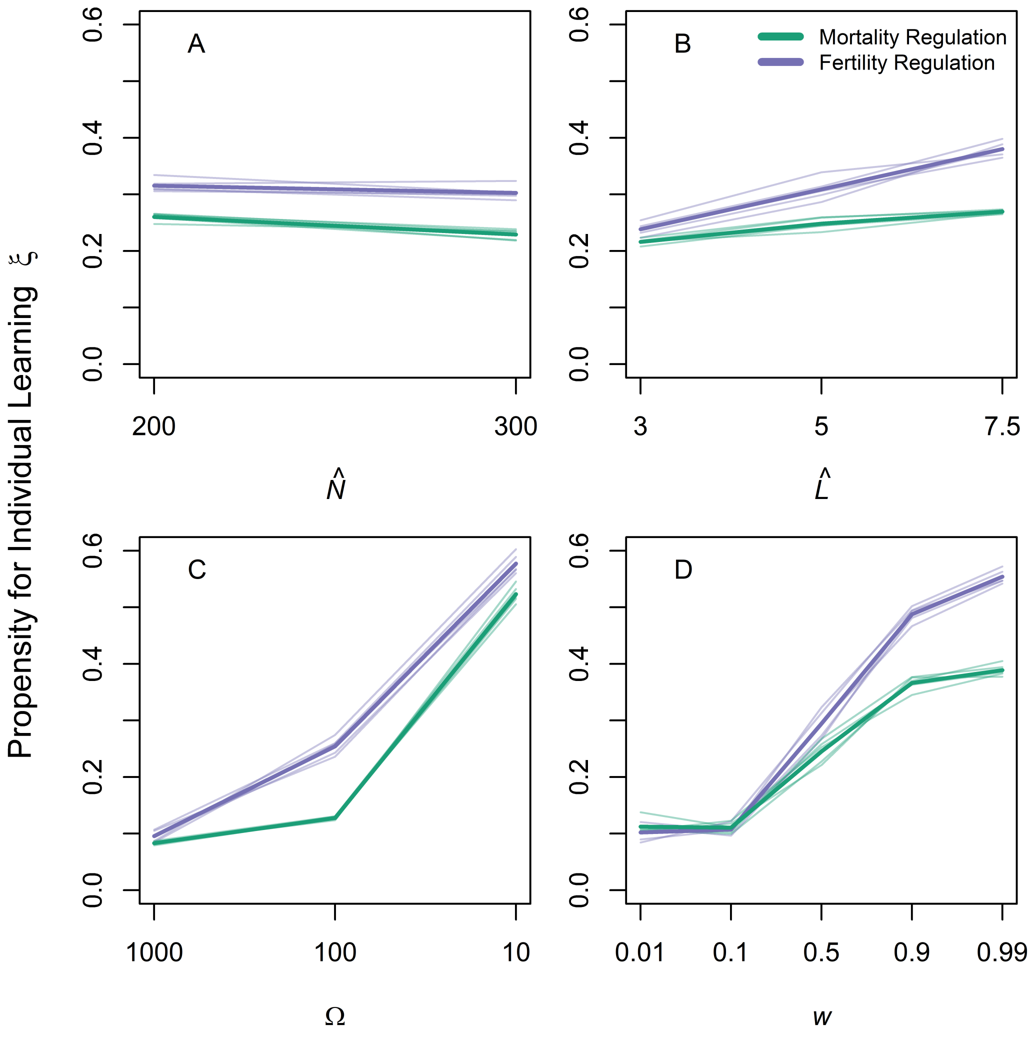

FIGURE S7. Results for simulations with strong population regulation ($\delta = \frac{1}{550}$). Average propensity for individual learning ξ as a function of (A) equilibrium population size $\hat{N}$ (values are based on baseline vital rates and thus correspond to situations when all individuals are not adapted); (B) expected life span $\hat{L}$ (values of 3, 5 and 7.5 years correspond to $s = 0.6\bar{6}$, $s = 0.8$ and $s = 0.8\bar{6}$, respectively) ; (C) expected time between environmental changes Ω ($= \frac{1}{u}$) and (D) success rate of individual learning. Transparent lines show results from 5 independent simulations, solid lines represent averages across different simulations. Results are averaged over all values of other parameters ($c = 0.95$).
